# Structural network topology and microstructural alterations of the anterior insula associate with cognitive and affective impairment in Parkinson’s disease

**DOI:** 10.1038/s41598-021-95638-8

**Published:** 2021-08-06

**Authors:** L. E. Jonkman, Y. Y. Fathy, H. W. Berendse, M. M. Schoonheim, W. D. J. van de Berg

**Affiliations:** 1 Department of Anatomy and Neurosciences, Amsterdam UMC, Vrije Universiteit Amsterdam, O|2 Building, De Boelelaan 1108 , 1081 HZ Amsterdam, The Netherlands; 2 Department of Neurology, Amsterdam UMC - location VUmc, Amsterdam, The Netherlands; 3 Department of Neurology, Erasmus Medical Center, PO Box 2040 3000 CA, Rotterdam, The Netherlands

**Keywords:** Parkinson's disease, Neuroscience

## Abstract

The aim of the current study was to assess the structural centrality and microstructural integrity of the cortical hubs of the salience network, the anterior insular cortex (AIC) subregions and anterior cingulate cortex (ACC), and their relationship to cognitive and affective impairment in PD. MRI of 53 PD patients and 15 age-matched controls included 3D-T1 for anatomical registration, and diffusion tensor imaging for probabilistic tractography. Network topological measures of eigenvector and betweenness centrality were calculated for ventral (vAI) and dorsal (dAI) AIC. Microstructural tract integrity between vAI, dAI and the ACC was quantified with fractional anisotrophy (FA) and mean diffusivity (MD). Structural integrity and connectivity were related to cognitive and affective scores. The dAI had significantly higher eigenvector centrality in PD than controls (*p* < 0.01), associated with higher depression scores (left dAI only, *r*_*s*_ = 0.28, *p* < 0.05). Tracts between dAI and ACC showed lower FA and higher MD in PD (*p* < 0.05), and associated with lower semantic fluency, working memory and executive functioning, and higher anxiety scores (range 0.002 < *p* < 0.05). This study provides evidence for clinically relevant structural damage to the cortical hubs of the salience network in PD, possibly due to extensive local neuropathology and loss of interconnecting AIC-ACC tracts.

## Introduction

Parkinson’s disease (PD) is one of the most common and debilitating neurodegenerative disorders in which motor, cognitive, and neuropsychiatric deficits are prevalent^1,2^. Although symptomatic treatments are available for motor symptoms, non-motor deficits remain poorly understood. About 83% of PD patients will develop some level of cognitive impairment^3^, the domains specifically affected include visuospatial, attention, executive, and even memory. In fact, about 10–20% of PD patients have MCI at time of diagnosis and up to 80% develop dementia provided they survive the disease for more than 10 years^4^. As such, these deficits have a huge impact on patients’ quality of life and social and mental well-being. However, to date, effective symptomatic or disease modifying treatments for cognitive impairment remain lacking. To meet the urgent need to find treatments delaying the onset or slowing cognitive impairment, more emphasis is needed on the substrates of cognitive impairment including but not limited to imaging biomarkers.

In PD, neuropathological lesions affect cortical and subcortical brain areas, as well as the integrity of interconnecting white matter tracts^5–7^. By this very nature, PD can be seen as a ‘disconnection syndrome’^8,9^. Critically, disconnection in PD was shown to be associated with cognitive and affective impairment^10–12^. Regional evaluations remain rare, but of interest.

The salience network, in which the insula and anterior cingulate cortex (ACC) play a key role, is involved in a myriad of cognitive, affective and viscero-sensory functions^13,14^. Several MRI studies indicate widespread involvement of the insular cortex in PD-related cognitive and affective decline, although in-vivo quantification of this damage remains difficult^15–19^. This difficulty partly lies in the heterogeneous nature of the insula, with a distinct cytoarchitecture of variably differentiated cortical layers, and widespread connectivity, which can be captured with diffusion MRI. The anterior insular cortex (AIC) consists of a transitional allocortical-neocortical dorsal anterior insula (dAI), and an allocortical ventral anterior insula (vAI)^20^. While the dAI is important in goal-oriented cognitive and executive control, the vAI plays a role in affective functions, in part due to its connections with limbic regions^21,22^. In turn, the posterior insula largely shows a granular 6-layer architecture^20^, and is involved in the integration of autonomic and interoceptive signals^22,23^.

Within the salience network, connections between the AIC and ACC are important for rapid orientation of attention and adaptive switching of cognitive control^13,21,24–26^. There is strong evidence that functional changes in the salience network are related to cognitive impairment and depression in PD, but structurally this network remains understudied^27,28^. Therefore, the aim of the current study is to address the structural centrality of the dAI and vAI, as well as the microstructural integrity of tracts between the dAI, vAI and ACC, in relation to cognitive and affective functions in PD patients and healthy controls. Revealing structural damage in these circuits would allow a more integrative view of non-moter deficits in PD, with a better understanding of the central role of the insula, its subregions and its connections.

## Results

### Subject demographics and clinical characteristics

The demographics and functional test scores are summarized in Table 1. PD patients had a mean age of 67.3 years (± standard deviation [SD] 6 years) with a moderate disease duration (mean 11.3 ± 3.6 years). Based on clinical scores, patients had mild to moderate disease severity (Unified Parkinson’s Disease Rating Scale [UPDRS] III median score of 31 (range 14–56) and Hoehn and Yahr scale [H&Y] median score of 2.5 (range 2–3)). There were no significant differences in age, sex distribution, or educational levels between PD patients and controls. With regards to cognitive assessment, Cambridge Cognitive Examination (CAMCOG) scores were significantly lower in PD patients compared to controls (t(63) = 4.54, *p* < 0.001). Beck Depression Inventory (BDI) and Beck Anxiety Inventory (BAI) test scores, as well as performance on additional cognitive tests, were significantly worse in PD compared to controls (*p* < 0.01). The Mini Mental Status Exam (MMSE) score was not significantly different between groups (control median = 28 (range 27–30); PD median = 28 (range 19–30), t(65) = 0.91, *p* = 0.336), although four PD patients fell below the MMSE score of 24.

**Table 1:** Subject demographics and functional test scores.

Demographics and functional tests		Controls (n = 15)		PD (n = 53)		*p* values
		N/count (%)	Median (range)	N/count (%)	Median (range)	
Age (Years)^		15	66.9 (52–81)	53	67.3 (54–81)	*p* = 0.85
Education	2	2 (13,3%)	5 (2–6)	16 (30,2%)	3 (2–6)	*p* = 0.07
	3	3 (20%)		15 (28,3%)		
	4	1 (6,7%)		2 (3,8%)		
	5	8 (53,3%)		19 (35,8%)		
	6	1 (6,7%)		1 (1,9%)		
Gender (Males)		66%	–	58%	–	*p* = 0.57
Disease duration (years)^		NA	NA	53	11.3 (4–21)	NA
UPDRS_III		NA	NA	53	31 (14–56)	NA
HY	2,0	NA	NA	21 (39,6%)	2.5 (2–3)	NA
	2,5	NA	NA	18 (34%)		NA
	3,0	NA	NA	14 (26,4%)		NA
Mini mental status exam		14	28 (27–30)	53	28 (19–30)	*p* = 0.34
CAMCOG_Total		14	99.5 (95–104)	53	96 (71–103)**	***p* < 0.001**
Semantic fluency		14	23.5 (16–40)	53	19 (5–32)*	***p* = 0.03**
Pattern recognition memory		14	23 (21–24)	52	21.5 (13–24)**	***p* < 0.001**
Spatial span length		14	5 (5–7)	52	5 (2–8)*	***p* = 0.01**
Spatial working memory		14	22 (0–57)	52	38 (2–106)*	***p* = 0.015**
Intra-extra dimension shift		14	9 (7–9)	51	9 (0–9)**	***p* = 0.003**
Vienna perseveration test		14	18 (14–27)	52	21 (10–63)*	***p* = 0.001**
Beck depression inventory		14	3.5 (0–23)	52	11 (0–31)**	***p* = 0.001**
Beck anxiety inventory		12	24 (21–31)	31	32 (24–57)**	***p* < 0.001**
LEDD		0		48	900 (225–2600)	NA

Education is based on ISCED system ranging from 0 to 6. CAMCOG_Total: Cambridge Cognitive Battery Total score, H&Y: Hoehn and Yahr stage, ISCED: International Standard Classification of Education, LEDD: Levodopa equivalent daily dose, PD: Parkinson’s disease, SD: Standard Deviation, UPDRS III: Unified Parkinson’s Disease Rating Scale part III, LEDD: L-DOPA equivalent daily dose.. ^mean and range is reported for these variables.. * *p* < 0.05, ** *p* < 0.01. Table adapted from Fathy et al., 2020^28^.

### Network topological differences of insula sub-regions

#### Eigenvector centrality

**Figure 1:**
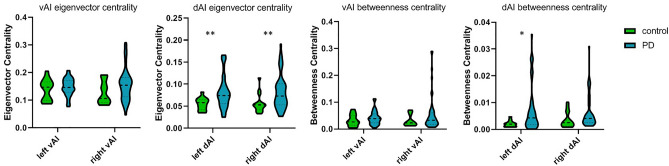
Group differences in eigenvector centrality and betweenness centrality. PD patients showed significantly higher left and right dAI eigenvector centrality and higher left dAI betweenness centrality than controls. vAI = ventral anterior insula; dAI = dorsal anterior insula. *p < 0.05 **p < 0.01 after Bonferroni correction.

The left dAI showed a significantly higher eigenvector centrality in PD than in controls (median = 0.07, interquartile range (IQR) = 0.056–0.096 vs median = 0.058, IQR = 0.042–0.065; *U* = 201 *p* = 0.004). The right dAI also showed a significantly higher eigenvector centrality in PD than controls (median = 0.07, IQR = 0.05–0.10 vs median = 0.05, IQR = 0.04–0.06, *U* = 217 *p* = 0.008). This was not significant for the left vAI (median = 0.14, IQR = 0.13–0.17 vs median = 0.14, IQR = 0.099–0.166, *U* = 344.5 *p* = 0.43) or right vAI (median = 0.15, IQR = 0.112–0.182 vs median = 0.11, IQR = 0.091–0.168, *U* = 291 *p* = 0.115) (Fig. 1).

Associating these significant increases in dAI eigenvector centrality to cognitive and affective measures in PD patients, an increase in left dAI centrality associated with higher scores on the BDI depression scale (*r*_*s*_ = 0.277; *p* = 0.047; Table 2). No significant associations were observed with any of the other clinical measures of cognitive and affective functions.

**Table 2:** Associations between centrality measures and cognitive and affective measures in PD.

	Left dAI eigenvector centrality	Right dAI eigenvector centrality	Left dAI betweenness centrality	Right dAI betweenness centrality
**CAMCOG**				
*r*_*s*_	− 0.081	0.098	− 0.045	0.199
*p-value*	0.564	0.487	0.749	0.153
N	53	53	53	53
**Fluency**				
*r*_*s*_	− 0.183	0.024	− 0.160	0.172
*p-value*	0.189	0.866	0.254	0.219
N	53	53	53	53
**PRM**				
*r*_*s*_	− 0.101	− 0.067	− 0.110	0.005
*p-value*	0.478	0.635	0.436	0.972
N	52	52	52	52
**SSP**				
*r*_*s*_	− 0.057	− 0.022	0.006	− 0.118
*p-value*	0.688	0.878	0.964	0.405
N	52	52	52	52
**SWM**				
*r*_*s*_	0.077	0.136	0.033	0.218
*p-value*	0.585	0.336	0.817	0.120
N	52	52	52	52
**IED**				
*r*_*s*_	− 0.059	− 0.156	− 0.076	− 0.055
*p-value*	0.682	0.274	0.595	0.700
N	51	51	51	51
**VPT**				
*r*_*s*_	0.262	− 0.112	0.223	− 0.078
*p-value*	0.061	0.431	0.111	0.584
N	52	52	52	52
**BDI**				
*r*_*s*_	0.277*	− 0.057	0.177	− 0.035
*p-value*	**0.047**	0.687	0.210	0.805
N	52	52	52	52
**BAI**				
*r*_*s*_	− 0.111	− 0.208	− 0.163	− 0.286
*p-value*	0.552	0.261	0.380	0.119
N	31	31	31	31

r_s_ = Spearman correlation; vAI = ventral anterior insula; dAI = dorsal anterior insula. CAMCOG = Cambridge Cognitive Examination revised test battery; PRM = pattern recognition memory; SSP = spatial span; SWM = spatial working memory; IED = the intra-extra dimensional set shift test; VPT = Vienna perseveration test; BAI = Beck Anxiety Inventory; BDI = the Beck Depression Inventory. *p < 0.05.

#### Normalized betweenness centrality

The left dAI showed a significantly higher betweenness centrality in PD than in controls (median = 0.004, IQR = 0.0018–0.0088 vs median = 0.002, IQR = 0.001–0.0026, *U* = 223 *p* = 0.01). The right dAI also showed a significantly higher betweenness centrality in PD than in controls (median = 0.004, IQR = 0.0025–0.0077 vs median = 0.0025, IQR = 0.0013–0.0066, *U* = 247 *p* = 0.026), but this did not survive multiple comparisons. No difference in betweenness centrality was found for the left vAI (median = 0.038, IQR = 0.023–0.056, *U* = 316 *p* = 0.228) or right vAI (median = 0.033, IQR = 0.018–0.061, *U* = 310 *p* = 0.193) (Fig. 1). There were no significant correlations between dAI betweenness centrality and cognitive and affective measures in PD patients. (Table 2).

### Microstructural alterations between dorsal anterior insula and ACC

**Figure 2:**
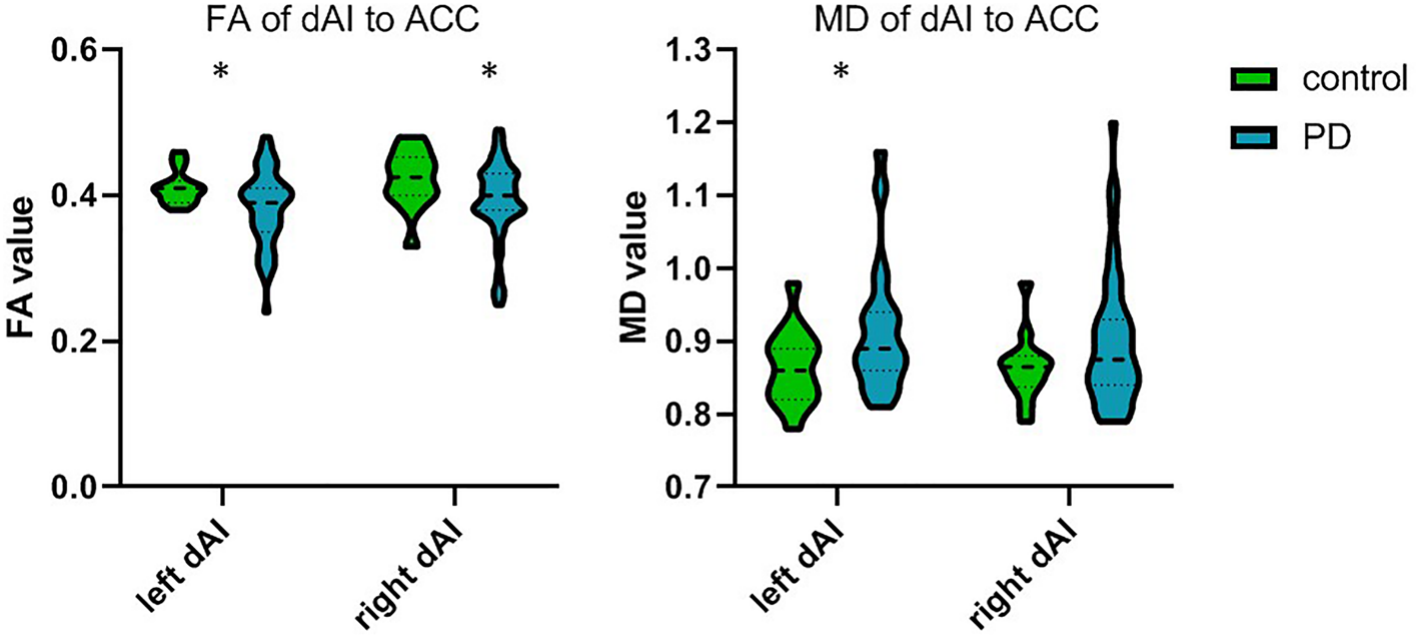
Group differences in FA and MD of dAI to ACC tract. PD patients showed lower FA in dAI to ACC tracts than controls, and higher MD in left dAI to ACC tract. FA = fractional anisotrophy; MD = mean diffusivity; vAI = ventral anterior insula; dAI = dorsal anterior insula. *p < 0.05.

The anterior insula has strong connections with the ACC^13^, and is associated with cognitive control in PD^28^. Since only the dAI, not the vAI, showed network topological alterations of eigenvector and betweenness centrality in PD, we assessed microstructural alteration in the connections between the dAI and ACC only. A significant lower FA was observed in PD patients within the tract between left dAI and left ACC compared to controls (median = 0.31, IQR = 0.035–0.041 vs median = 0.41, IQR = 0.39–0.042, *U* = 244 *p* = 0.040), as well as a significant higher MD (median = 0.89, IQR = 0.86–0.94 vs median = 0.86, IQR = 0.82–0.89, *U* = 238 *p* = 0.040) Lastly, a significantly lower FA was observed in the tract between right dAI and right ACC in PD compared to controls (median = 0.40, IQR 0.38–0.4, *U* = 235 *p* = 0.034), but here MD was not significant (median = 0.88, IQR = 0.84–0.93, *U* = 292 *p* = 0.254) (Fig. 2).

Associating microstructural alterations of the tracts between dAI and ACC to cognitive and affective measures in PD, significant associations for increased MD between the left and right dAI and ACC were found for semantic fluency (left *r*_*s*_ = -0.31, *p* = 0.028; right *r*_*s*_ = -0.28, *p* = 0.04), spatial span length (SSP; left *r*_*s*_ = -0.30, *p* = 0.036; right *r*_*s*_ = -0.28, *p* = 0.042) and spatial working memory (SWM; left *r*_*s*_ = 0.33, *p* = 0.02; right *r*_*s*_ = 0.43, *p* = 0.002). In addition, a significant association was observed between increased MD of the left dAI and ACC tract and increased BAI (*r*_*s*_ = 0.39, *p* = 0.03) (Table 3).

**Table 3:** dAI-ACC WM tract microstructural feature association with cognitive and affective measures in PD.

	FA of left dAI to ACC	FA of right dAI to ACC	MD of left dAI to ACC	MD of right dAI to ACC
**CAMCOG**				
*r*_*s*_	0.135	0.007	− 0.277	− 0.267
*p-value*	0.350	0.959	0.054	0.056
N	50	53	49	52
**Fluency**				
*r*_*s*_	0.137	− 0.057	− 0.314*	− 0.280*
*p-value*	0.341	0.687	**0.028**	**0.044**
N	50	53	49	52
**PRM**				
*r*_*s*_	− 0.160	− 0.182	− 0.107	− 0.007
*p-value*	0.271	0.196	0.468	0.963
N	49	52	48	51
**SSP**				
*r*_*s*_	0.168	0.184	− 0.304*	− 0.285*
*p-value*	0.249	0.192	**0.036**	**0.042**
N	49	52	48	51
**SWM**				
*r*_*s*_	− 0.144	− 0.083	0.329*	0.429**
*p-value*	0.324	0.556	**0.022**	**0.002**
N	49	52	48	51
**IED**				
*r*_*s*_	0.027	0.164	− 0.144	− 0.129
*p-value*	0.858	0.252	0.335	0.371
N	48	51	47	50
**VPT**				
*r*_*s*_	− 0.014	− 0.060	0.089	0.104
*p-value*	0.925	0.671	0.548	0.468
N	49	52	48	51
**BDI**				
*r*_*s*_	− 0.212	− 0.110	0.214	0.263
*p-value*	0.144	0.436	0.143	0.062
N	49	52	48	51
**BAI**				
*r*_*s*_	− 0.298	0.014	0.392*	0.289
*p-value*	0.104	0.940	**0.032**	0.121
N	31	31	30	30

r_s_ = Spearman correlation; vAI = ventral anterior insula; dAI = dorsal anterior insula. CAMCOG = CAMbridge COGnitive examination revised test battery; PRM = pattern recognition memory; SSP = spatial span; SWM = spatial working memory; IED = the intra-extra dimensional set shift test; VPT = Vienna perseveration test; BAI = Beck Anxiety Inventory; BDI = the Beck Depression Inventory. *p < 0.05, **p < 0.01.

## Discussion

The salience network, with the insula and ACC as its main cortical hubs^26^, shows structural and functional alterations associated with non-motor deficits in PD^15,27,29,30^. In these studies, the (anterior) insula is mostly studied as one region, although the cytoarchitecture of the anterior insula reveals a sub-regional division into dorsal and ventral anterior insula (dAI and vAI), associated with cognitive control and affective functions, respectively^20,21,23^. Our study shows that the dAI has a higher betweenness and eigenvector centrality in PD, the latter being associated with depression. In addition, white matter tracts between the dAI and ACC showed microstructural damage in PD, associated with cognitive impairments in a range of domains related to semantic fluency, working memory capacity, executive functioning and with symptoms of anxiety.

Other nodes in the salience network include the amygdala, hypothalamus, ventral striatium, thalamus and several brainstem nuclei^21^. However, it is the anterior insula and ACC within this network that receive the most attention in PD. The anterior insula and ACC are strikingly similar in their difference to other cortical regions; both regions contain subregions featuring agranular cortices (without a visible layer 4), and both regions contain von Economo neurons, unique cells thought to play a role in awareness^31^. In addition, the (anterior) insula and ACC are severely affected by Lewy body pathology in advanced stages of PD, during which patients often suffer from cognitive impairment^32^.

Network topological measures, specifically of centrality, have consistently shown alterations suggesting network disintergration in PD, and associate with (onset of mild) cognitive impairment^33–35^. This is in line with our results showing higher eigenvector and betweenness centrality of the dAI in PD, indicating a more central role for these regions, possibly due to a loss of connections with non-hub regions^36^. Furthermore, altered eigenvector centrality of the dAI shows an association with depressive symptoms, which ranged from mild to moderate in our PD cohort. This association is consistent with the literature in which disrupted anterior insula connectivity and white matter atrophy have been shown to contribute to depression associated with PD^30,37^.

In addition to a change in insular network topology, we specifically addressed microstructural alterations of the white matter tracts between the dAI and ACC. The dAI is strongly connected to other regions important for cognitive control, most notably the ACC^21,26,38^. Our results show microstructural damage within this tract, specifically a decrease in FA and increase in MD, which may relate to the increasing pathological load in both regions. It has been shown that within the insular cortex, especially the anterior insula subregion is susceptible to severe Lewy body pathology in PD^39^. Furthermore, the increased MD of the dAI to ACC bundles was associated with a decline in semantic fluency, working memory, executive functioning, and an increase in symptoms of anxiety, reflecting the wide array of cognitive and affective domains associated with insular (dys)function^15,40^.

The main strength of our study is the region-specific approach, based on functional associations within the salience network, specifically the anterior insula and ACC^28^. There are however, several limitations that need to be addressed. First, our retrospective cohort consisted of a unbalanced and limited sample size, particularly for controls, and although comparable in size to other insula studies, this limits the generalizability of our data, specifically in terms of effects of normal aging on structural integrity. Larger cohorts should further explore these effects as well as compare with dementia syndromes. Second, future endeavors should investigate the impact of covariates in group differences and association with cognitive outcome measures. Especially sex has recently shown to have a differential effect on patterns of cognitive decline in PD^41^. Furthermore, because the anterior insula is involved in such a myriad of cognitive and affective functions, including speech, attention, saliency, and emotional processing^13^, we included all available cognitive tests, limiting our ability to correct for multiple comparisons in the associations. All in all, further research in a large cohort of PD patients is needed to further establish structural connectivity alterations of the (anterior) insular cortex with ACC, and other subcortical brain regions in the salience network, associated with specific cognitive domains in PD.

## Conclusions

In this study, we found alterations in network topology and microstructural integrity of white matter tracts for the anterior insula, primarily centered around the dAI, which showed disconnection from the ACC. These alterations within the cortical hubs of the salience network were associated with PD related cognitive impairment and affective symptoms. Considering the two regions’ functional importance in cognitive control and their susceptibility to neuropathological change, the changes in network topological and microstructural integrity found in the current study adds to an integrative view of these regions, and towards a more precise understanding of cognitive impairment in PD.

## Material and methods

### Standard protocol approvals, registrations, and donor consents

The research protocol was approved by the local institutional ethics review board of Amsterdam UMC, location VUmc (Amsterdam, the Netherlands), in adherence to the Helsinki declaration. All participants provided written informed consent to participate in the study. Clinical datasets and MRI imaging of both PD patients and healthy individuals were collected in a standardized manner at the outpatient clinic of Amsterdam UMC, location VUmc, as previously described^42^.

### Inclusion of participants

PD patients fulfilled the UK Parkinson’s Disease Brain Bank Clinical diagnostic criteria^43^. Age-of-onset and disease duration was calculated based on the first occurrence of PD motor symptoms. Educational levels were classified according to the International Standard Classification of Education with scores ranging from 0–6 (0 = no primary education & 6 = university education). Exclusion criteria for the study included previous stereotactic surgery, extensive white matter lesions (Fazekas > 2)^44^, as well as other abnormalities (e.g. space occupying lesions) seen on structural MRI images. Subjects were examined by a trained neurologist with specific experience in movement disorders. Healthy volunteers were recruited as either spouses of patients or non-neurological participants within the same age range. After excluding two patients with insular hypo-intensities on T1 MRI (suspected of being infarcts), we included data of 53 sporadic PD patients and 15 age-matched controls for whom complete clinical, neuropsychological, and imaging data were available^28^. Clinical and imaging evaluations, as previously described^42,45^, were performed on the same day in the “ON” medication state to avoid unnecessary discomfort.

### Clinical, cognitive, and neuropsychiatric assessment

The Unified Parkinson’s Disease Rating Scale part III (UPDRS-III) was used to assess the severity of motor symptoms^46^. Hoehn and Yahr (H&Y) staging was used to provide information on patients’ disease stages^47^. PD patients were on combinations of anti-parkinsonian medications, An L-DOPA equivalent daily dose (LEDD) was calculated for each patient^48^ (Table 1). Cognitive performance was evaluated using the Mini Mental Status Examination (MMSE) and the CAMbridge COGnitive examination revised test battery (CAMCOG). This latter assesses orientation (maximum score: 10), language (maximum score: 30), memory (maximum score: 27), attention and calculation (maximum score: 9), praxis (maximum score: 12), abstract thinking (maximum score: 8), and perception (maximum score: 9)^49^. Other cognitive tests included in the study to assess working memory, visuospatial memory, attention and executive functions consist of semantic fluency, pattern recognition memory (PRM), spatial span (SSP), spatial working memory (SWM), the intra-extra dimensional set shift test (IED), and the Vienna perseveration test (VPT). As the insular cortex is not only involved in cognition, but also in affective alterations in PD^50–52^, symptoms of anxiety and depression were assessed through the Beck Anxiety inventory (BAI) and Beck Depression Inventory (BDI), respectively.

### MRI acquisition

All participants underwent structural whole-brain 3 T MR imaging (Sigma HDXT, V15M; GE Healthcare) using a sagittal three-dimensional T1-weighted fast spoiled gradient-echo sequence (repetition time [TR] = 7.8 ms, echo time [TE] = 3.0 ms, inversion time [TI] = 450 ms; flip angle 12°; resolution 1.0 × 0.9 × 0.9 mm). In addition, DTI acquisition consisted of five volumes without directional weighting, and 30 volumes with non-collinear gradient directions (b-value 1000 s/mm^2^, TR = 13,275 ms, TE = 91 ms, slice thickness 2.4 mm; in-plane resolution 2 × 2 mm).

### Image pre-processing

Data preprocessing was performed using tools from the FMRIB (Functional Magnetic Resonance Imaging of the Brain) software library (FSL 5.0.9, http://www.fmrib.ox.ac.uk/fsl). For 3D-T1 images, brain extraction (bet functionality) and grey and white tissue-type segmentation with partial volume estimation was carried out (SIENAX functionality)^53^. The diffusion-weighted images were corrected for motion and eddy current distortion using FMRIB’s Diffusion Toolbox (FSL-FDT; part of FSL 5.0.9). Non-linear registration (FNIRT) was used to calculate registration parameters to standard space, which were inverted to bring our anatomical atlas (see below) into subject anatomical space, using nearest neighbor interpolation. The full atlas was then multiplied with the SIENAX grey matter mask, to remove CSF and eliminate displacement due to grey matter atrophy.

The transformation between 3DT1 and diffusion-weighted images was derived by using Boundary-Based registration (BBR; part of FSL). Bedpostx was run to build up diffusion parameter distributions at each voxel.

### Construction of an anatomical brain atlas with insula sub-regions

A surface‐based version of the automated anatomical labeling (AAL) atlas was used to parcellate the cortex into 78 areas^54^ and FIRST (part of FSL) was used to delineate deep gray matter structures. We did not use the insular cortex from this atlas, as it did not provide an anterior insula sub-division. Instead, delineation of the insular cortex was based on the Hammersmith brain atlas^55^. Based on the anatomical features of the insular cortex as described in Naidich et al.^56^ and shown in a previous publication by our group^28^, the three short gyri regions were merged into a single region designated as the dorsal anterior insula (dAI). The anterior inferior cortex will be referred to as the ventral anterior insula (vAI). As our focus is on cognition rather than autonomic dysfunction and interception, we only included the anterior and not posterior insula in our atlas. In summary, the constructed anatomical atlas consisted of a total of 94 nodes: 90 (sub)cortical areas (excluding insula), as well as the left and right dAI and vAI.

### Construction of structural connectomes and computation of graph theory measures

Probabilistic tractography was conducted (probtrackx2 wit standard settings, 5000 streamlines per voxel) to obtain probabilistic maps of WM connections running between all pairs of nodes (regions of interests (ROIs)) of the anatomical atlas. Each ROI was overlaid on the probalistic map to obtain the number of fibers at a node, subsequently corrected for node volume, resulting in a non-weighted 94 × 94 structural network matrix for each subject.

The structural network matrices were analyzed in Matlab (MATLAB R2012a, The MathWorks Inc., Natick, MA, 2000). The matrix was symmetrized by computing the average between the original matrix and its transpose, and subsequently thresholded to reduce the number of false-positive connections; the top 20% strongest links were retained^57^. From the binarized matrix, graph theoretical characteristics were computed using the Brain Connectivity Toolbox as described previously^58^.

In the current study, we included eigenvector centrality and normalized betweenness centrality of the dAI and vAI. Eigenvector centrality is defined as a self-referential measure of centrality; nodes (e.g. dAI or vAI) have a high eigenvector centrality if they connect to other nodes in the network that have a high eigenvector centrality^58^. Normalized betweenness centrality (BC) is defined as the fraction of all shortest paths in the network that contain the dAI or vAI, divided by the number of nodes (N) in the network; BC/[(N-1)(N-2)]. This normalizes the Betweenness centrality to the range [0,1], with larger values corresponding to greater centrality^58^.

### Assessment of microstructural tract integrity between anterior insula and ACC

**Figure 3:**
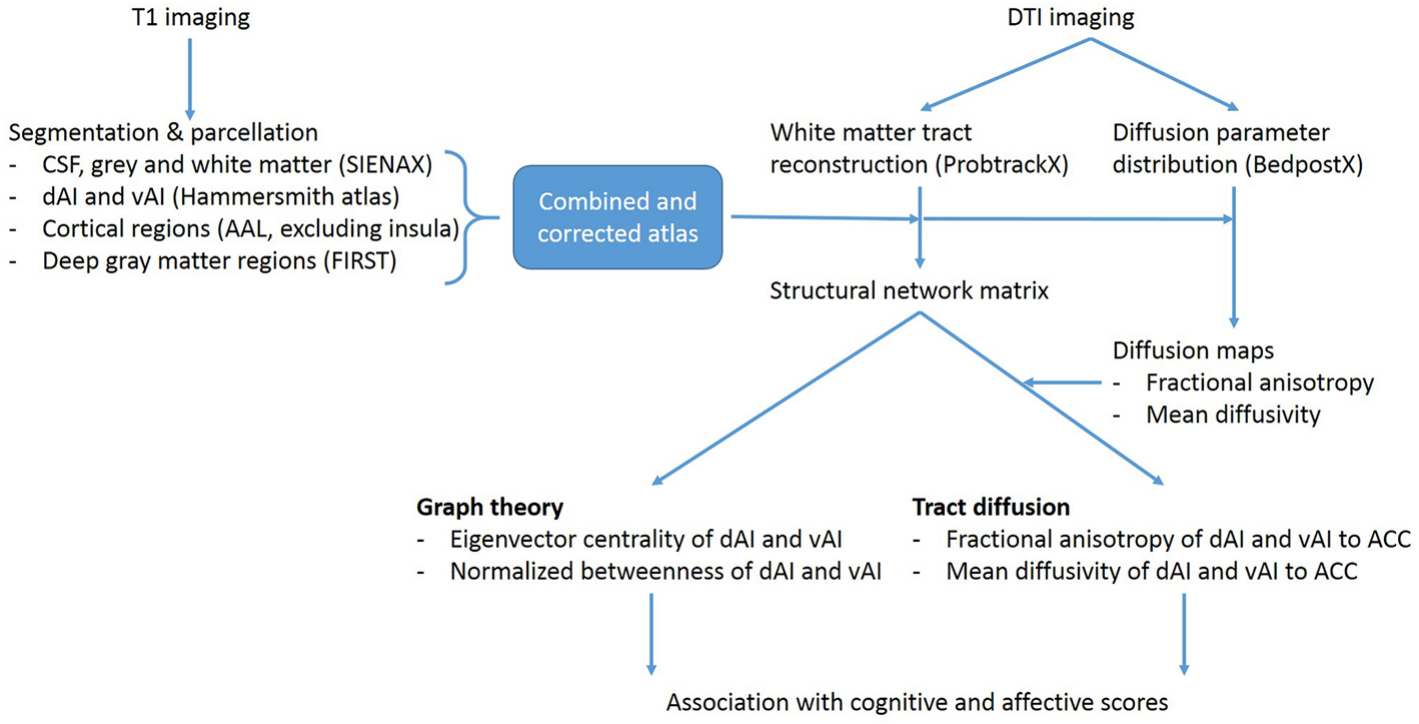
Flowchart of MRI methods and outcome measures. From the T1 images an atlas is constructed which includes the (dorsal and ventral) anterior insula sub-regions from the Hammersmith atlas, as well as other cortical and subcortical regions from the AAL atlas, together corrected for CSF and displacement due to gray matter atrophy. From the DTI images, the diffusion parameters were built, resulting in an FA and MD map. In addition, white matter tracts were reconstructed from the DTI images, and combined with the constructed atlas, the structural network matrix was created. From this, the graph theoretical outcome measures (EC and BC) could be calculated, as well as the microstructural diffusion (FA and MD) outcome measures of specific anterior insula to anterior cingulate tracts. These outcome measures were then compared between PD patients and controls, as well as associated with cognitive and affective scores in PD patients. DTI = diffusion tensor imaging; EC = eigenvector centrality; BC = betweenness centrality; FA = fractional anisotropy; MD = mean diffusivity.

Aside from obtaining graph theoretical measures of the AIC sub-divisions, a restricted structural connectivity analysis was performed between the dAI, vAI and the ACC, to obtain the microstructural integrity of the tracts connecting these regions. The dAI and vAI were entered as a way-point, and the ACC as an end-point, in order to provide a map of all white-matter voxels included in the tract. These connectivity maps were subsequently thresholded and binarized, and used to calculate the mean fractional anisotropy (FA) and mean diffusivity (MD) of the tract by overlaying the tracts onto the diffusion maps. Tracts and diffusion overlays were visually inspected, which resulted in the exclusion of one PD patient for this analysis, which showed erroneous tracts going through the CSF. For an overview of our processing pipeline, see Fig. 3.

### Statistical analysis

Descriptive and statistical analyses were performed using IBM SPSS 22.0 for Windows (SPSS, Inc., Chicago, IL, USA). Normally distributed continuous demographic data were assessed using an independent two-sample t-test. Categorical variables were analyzed using Chi-square tests. Most MRI outcome measures, cognitive and affective scores, as well as their residuals, were not normally distributed. Therefore, we continued our analysis with non-parametric tests, corrected for multiple comparisons. Differences between PD and controls for network topology (e.g. eigenvector centrality and normalized betweenness centrality), were assessed with non-parametric Mann–Whitney U test and Bonferroni corrected for multiple (four) comparisons; left as well as right dAI and vAI. Similarly, microstructural tract integrity of insular sub-regions to the ACC, were assessed with non-parametric Mann–Whitney U test and Bonferroni corrected for multiple (four) comparisons; left as well as right dAI and vAI to ACC. Correlations of microstructural integrity of tracts with cognitive and affective test scores in PD patients were done using non-parametric Spearman’s correlation coefficients.

## Data availability

The datasets generated during and/or analysed during the current study are available from the corresponding author on reasonable request.
